# Correlation study of BDNF/TrkB/CREB, violence, and cognitive function in first-episode drug-naive schizophrenia patients

**DOI:** 10.3389/fpsyt.2025.1586613

**Published:** 2025-06-04

**Authors:** Tiankai Jiang, Zhipeng Li, Tao Yu, Xudong Zhou, Tiantian Jiang, Yuhang Liang, Chen Yu, Min Zhu, Wenyu Wu

**Affiliations:** Department of Clinical Psychology, Mental Hospital of Yunnan Province, Kunming, Yunnan, China

**Keywords:** schizophrenia, BDNF, TrkB, CREB, violence, cognition

## Abstract

**Introduction:**

Schizophrenia (SCZ) is a complex mental disorder affecting thought processes, perceptions, and emotional regulation.

**Methods:**

This study investigated the relationship between brain-derived neurotrophic factor (BDNF), tropomyosin receptor kinase B (TrkB), and cAMP response element-binding protein (CREB) expression with violence risk and cognitive function in first-episode, drug-naive SCZ patients. We recruited 62 SCZ patients and 62 healthy controls from the Affiliated Mental Health Center of Kunming Medical University. Sociodemographic data and psychopathological measures were collected. qRT-PCR and Western blotting assessed BDNF, TrkB, and CREB expression. Cognitive function and violence risk were evaluated using the Positive and Negative Syndrome Scale (PANSS), History of Violence, Clinical, Risk Assessment Scale (HCR-20), Modified Wisconsin Card Sorting Test (M-WCST), and Wechsler Memory Scale (WMS).

**Results:**

Correlation and regression analyses explored relationships between signaling factors and clinical measures. SCZ patients exhibited significantly lower BDNF, TrkB, and CREB levels than controls, higher HCR-20 scores, and impaired cognitive performance. BDNF negatively correlated with HCR-20 scores and positively with nonpreservative errors. CREB positively correlated with memory quotient. Multivariate linear regression suggested CREB plays a crucial role in both violent behavior and cognitive function in SCZ patients. Principal component analysis (PCA) combined highly correlated P_score, N_score, and PANSS_total into one principal component PC1, with logistic regression identifying PC1 as an associated factor for violence. A model incorporating BDNF, TrkB, and CREB predicted SCZ with an area under the ROC curve of 0.733.

**Discussion:**

Our findings suggest CREB plays a key role in SCZ-related violence and cognition, while BDNF, TrkB, and CREB may serve as predictive markers and potential therapeutic targets.

## 1 Introduction

Schizophrenia (SCZ) is a mental illness with a global prevalence of about 0.45% (1). It manifests a range of symptoms, including negative symptoms (such as social withdrawal and aversion), positive symptoms (such as hallucinations, delusions, and disorganized thinking), and cognitive dysfunction (1). While the exact cause of SCZ remains unclear, it is widely regarded as the result of an interplay of genetic, biological, environmental, and psychological factors (2). The primary treatment approaches for SCZ include antipsychotic medications and psychotherapy, particularly cognitive-behavioral therapy (2).

Among its symptoms, cognitive decline is a core feature of SCZ, playing a crucial role in determining patient prognosis and functional outcomes. This cognitive impairment often emerges early in the disease course and tends to worsen over time (3). Major cognitive areas, working memory, attention, executive function, and processing speed, are significantly disrupted in individuals with SCZ (4). These cognitive deficits are the primary contributors to disability among SCZ patients (5). Therefore, improving cognitive deficits in patients with SCZ is a top priority in treatment.

Meanwhile, individuals with SCZ are at an increased risk of various adverse outcomes, such as violent behavior, compared to the general population (6). This population is at a higher risk of violence compared to the general population, though most of them never exhibit violent behavior during lifetime (7). A meta-analysis by Fazel et al. demonstrated a four-fold increase in the risk of violent crime among men with SCZ and an eight-fold increase among women compared to the general population (8). Psychiatric symptoms, cognitive impairments, and the potential violent behavior leads to reduced academic and professional performance, as well as a diminished quality of life, placing a heavy burden on patients, their families, and society. Therefore, identifying the risk of violence and implementing strategies to reduce violent behavior are critical goals in SCZ treatment.

Due to the close association between cognitive impairment and nerve damage, brain-derived neurotrophic factor (BDNF) is essential for neuronal growth and synaptic plasticity (9, 10). In this context, notably, the receptor of BDNF, tropomyosin receptor kinase B (TrkB), and cAMP response element-binding protein (CREB) have emerged as critical parts. A previous study have highlighted the importance of BDNF/TrkB/CREB signaling pathway in ameliorating neuronal damage and cognitive impairment (11). BDNF is vital in neuroplasticity, which influences the survival, growth, and differentiation of neurons (12). The interaction between BDNF and TrkB is essential for mediating the effects of BDNF on synaptic plasticity, which is fundamental in learning and memory processes (13). Emerging evidence suggests that alterations in BDNF levels may correlate with various psychopathological conditions, such as depression (14), SCZ (15), and anxiety disorders (16). CREB is a transcription factor that plays a key role in mediating BDNF signaling (17), and the dysregulation of neuroplasticity and memory processes might alter CREB expression (18). Research found that CREB may be a potential therapeutic target for SCZ (19). We noticed that the expression of BDNF, TrkB, and CREB were generally reduced in patients with SCZ (11), which may lead to impaired neuroplasticity, which in turn affects cognitive function and increases the risk of violence.

Therefore, in this study, we first examined mRNA and protein levels of BDNF, TrkB, and CREB using qRT-PCR and western blotting in first-episode, drug-naive patients with SCZ and controls. We then explored the associations of these factors with cognitive function and violent behavior. To determine whether these signaling factors, along with demographic and psychopathological characteristics, influenced the risk of violence and cognitive performance, multivariate linear regression was employed. Additionally, multivariate logistic regression was utilized to identify potential predictors of violent behavior in SCZ patients. Ultimately, we discussed the potential of using a logistic regression model incorporating BDNF, TrkB, and CREB to predict SCZ. This study provides an important theoretical basis for understanding the pathophysiological mechanisms and clinical manifestations of SCZ.

## 2 Materials and methods

### 2.1 Study population

This study was approved by the Institutional Review Board of the Affiliated Mental Health Center of Kunming Medical University, and all the study procedures were in accordance with the Declaration of Helsinki. A total of 62 inpatients and outpatients with SCZ and 62 healthy individuals were recruited from this institute between 2022 and 2024.The healthy controls were matched with the SCZ group in terms of sex, age, and education.

The inclusion criteria of SCZ were as follows:

1. Aged≥18 years, male or female;
2. Met the diagnosis of SCZ according to the International Statistical Classification of Diseases and Related Health Problems 10th Revision (ICD-10);
3. No prior treatment with antipsychotic medications;
4. Ability to understand the content of the study, participate voluntarily, and cooperate with the evaluation;
5. Informed consent signed by the patient’s guardian.

The inclusion criteria of control group were as follows:

1. Having no history of mental disorders, family history of mental disorders;
2. Male or female, aged≥18;
3. Good compliance and ability to cooperate with the completion of relevant inspections;
4. Signed informed consent.

The exclusion criteria were as follows:

1. Inability cooperate or complete the assessment;
2. Comorbid mental disorders;
3. Severe physical illness or critical healthy state.

### 2.2 Blood sample collection

To minimize variability, participants were instructed to avoid emotional stress and strenuous physical activity prior to blood collection. After an overnight fast, 5 mL of peripheral venous blood was collected from each participant between 7:00 and 9:00 AM. Blood samples were drawn using EDTA-anticoagulated vacuum blood collection tubes. The collected blood samples were stored at 4°C for 4 h before processing. Samples were then centrifuged at 1500 g for 15 min at low speed to separate the plasma. Subsequently, 1 mL of PBS was added to the residual blood in the collection tube for dilution purposes.

For lymphocyte isolation, 3 to 5 mL of lymphocyte separation medium was placed in a new centrifuge tube, and the diluted blood was carefully layered on top, ensuring a clear interface between the two layers. The samples were centrifuged at room temperature at 500–1000 g for 20 to 30 min using a horizontal rotor. After centrifugation, the buffy coat layer was carefully aspirated and transferred into two clean 1.5 mL EP tubes. These samples were then centrifuged at 250 g for 10 min. The supernatant was discarded, and the cell pellets were resuspended in PBS and centrifuged again at 250 g for 10 min. After the final centrifugation, the supernatant was carefully removed, and the isolated lymphocytes were stored at -80°C for future analysis.

### 2.3 Quantitative real-time PCR

Followed by thorough mixing through repeated pipetting to ensure complete homogenization, Lymphocytes were treated with 1 ml of Trizol. The samples were then incubated at 4°C for 15 min to facilitate cell lysis. Subsequently, chloroform was added at a ratio of 1:5 relative to the Trizol volume, and the mixture was vigorously shaken until a stable emulsion formed. The samples were incubated again at 4°C for 15 min. Centrifugation was performed at 12,000 rpm for 15 min at 4°C, after which the upper aqueous phase was carefully aspirated and transferred into a fresh 1.5 mL EP tube. An equal volume of isopropanol was added, and the mixture was gently homogenized and incubated at 4°C for an additional 15 min. After the second centrifugation at 12,000 rpm for 15 min at 4°C, the supernatant was discarded. The RNA pellet was washed with 1 mL of 75% ethanol, and then centrifugated at 7,500 rpm for 5 min at 4°C. The RNA pellet was air-dried at room temperature and subsequently dissolved in an appropriate volume of RNase-free water based on the yield of RNA. Reverse transcription was then performed to synthesize cDNA, which was used in quantitative PCR (qPCR) assays to quantify the mRNA levels of BDNF, TrkB, and CREB. The qRT-PCR analysis included 62 patient samples and 62 control samples. The following sequences of the primers were used for qPCR, which were shown in **Supplementary Table S1**. Fold changes of expression levels were calculated by the 2−ΔCt method.

### 2.4 Western blotting

Cultured lymphocytes were dissolved in RIPA buffer (Beyotime Biotechnology) containing PMSF protease inhibitor for the extraction of total protein, and lysed at 4°C for 30 min. The protein quantification was then measured by BCA Protein Assay Kit (Beyotime Biotechnology). Proteins (30 µg) were exposed to 10% SDS-PAGE and electroblotted onto a PVDF membrane (EMD Millipore, Bedford, MA, USA). The membranes were incubated overnight at 4°C with indicated primary antibodies (BDNF (1:1000, cat.# ab205067;Abcam), TrkB (1:1000, cat.# ab134155;Abcam) and CREB (1:1000, cat.# ab32515;Abcam), GAPDH (1: 1000, cat.#10494-1-AP;Proteintech)). Secondary antibodies were subsequently used for further incubation at 25°C for 1 h, which was diluted at 1:2000. The information of the antibodies was shown in **Supplementary Table S1**. For quantification, ImageJ software was used to measure the grayscale intensity of each protein band. The expression level of each target protein (BDNF, TrkB, and CREB) was normalized to the intensity of the corresponding GAPDH band within the same lane to correct for loading differences. The relative protein expression was then calculated as the ratio of target protein intensity to GAPDH intensity. These normalized values were used for statistical comparisons between groups.

Due to sample quality issues—such as limited volume or RNA/protein degradation during storage—a subset of samples failed to meet the quality control criteria and were excluded. Importantly, these exclusions were based solely on objective quality control criteria and without systematic bias. As a result, Western blot analysis was conducted on 40 participants out of the total 62 in each group. All included samples met strict quality thresholds to ensure the reliability of protein measurements.

### 2.5 Assessments

#### 2.5.1 Negative and positive symptoms

Psychopathology was assessed using the Positive and Negative Syndrome Scale (PANSS) (1991). PANSS evaluates the severity of positive and negative symptoms in patients with schizophrenia, and provided a quantitative measure for clinical research and treatment outcome assessments. It consisted of 30 items and divided into three main dimensions: (1) Positive Symptoms (7 items): assessing symptoms such as hallucinations, delusions, and thought disorder; (2) Negative Symptoms (7 items): assessing symptoms such as flat affect, social withdrawal, and lack of motivation. (3) General Psychopathology: (16 items): assessing other psychological symptoms such as anxiety, depression, and hostility. Each item is rated on a scale from 1 to 7 (1: Absent; 2: Minimal; 3: Moderate; 4: Moderate-severe; 5: Severe; 6: Extreme; 7: Extreme severity). In this study, the scores for all items were used as dependent measures.

#### 2.5.2 Assessment of violence

The Historical Clinical Risk Management-20 (HCR-20) (20) [German version: (21)] is a widely used structured professional judgment (SPJ) instrument for the assessment of risk for violent behavior. The tool comprises 20 static and dynamic variables, divided into ten historical (e.g., previous violence, young age at first violent incident, employment problems), five clinical (e.g., lack of insight, negative attitudes, impulsivity), and five risk management factors (e.g., lack of personal support, non-compliance with remediation attempts, stress).

Each item is scored on a 3-point scale (0–2), and the rater assigns a structured final risk judgment (low, medium, or high). The instrument demonstrates good concurrent validity (22) and moderates to strong predictive accuracy (23, 24) in offender populations. In this study, the SCZ and control groups were assessed with the HCR-20 second version. Participants classified as aggressive according to clinical criteria and scoring ≥ 21 on HCR-20 were assigned to violent group. Those considered nonaggressive based on clinical criteria and scoring ≤ 20 on HCR-20 were categorized as the nonviolent group.

#### 2.5.3 Cognitive functioning

The Modified Wisconsin Card Sorting Test (M-WCST) (25) is a neuropsychological assessment tool used to evaluate cognitive flexibility, abstract reasoning, and the ability to shift cognitive strategies in response to changing environmental conditions. The WCST is designed to assess an individual’s ability to organize information, shift cognitive strategies, and adapt to changing rules. It is commonly used in clinical settings to evaluate patients with various neurological and psychiatric disorders.

The test consists of 4 key cards and 48 response cards. Each card depicts figures that vary in color, shape, and number. The participant is required to sort these cards according to specific rules that change during the test.

Seven indices are calculated for the M-WCST:

Correct response (CR); Number of trials to complete the first category (TCFC); Number of completed categories (CC); Total errors (TE); Preservative errors (PE); Non-preservative errors (NPE); Time for task completion (s).

The assessment was performed by the administration of the Wechsler Memory Scale (WMS), with minor revised by Gong Yaoxian et al. (26). The WMS is a set of memory test scales commonly used at present, which can detect a variety of memory functions. In our study, the counting, picture recall, visual recognition, and reciting numbers in ascending or descending order were selected. The count including 1-100, 100–1 assesses long-term memory. Picture recall and visual recognition assesses short-term memory. Memorizing numbers assesses instantaneous memory.

The total scores for each subtest are summed, and the memory quotient (MQ) is calculated based on the weighted total score. The MQ is used to measure an individual’s memory and information processing ability. It uses a series of memory tests to assess an individual’s performance in areas such as short-term memory, and long-term memory.

### 2.6 Statistical analysis

Statistical analysis was conducted using the R package. Demographic and clinical variables were compared between groups by nonparametric Mann-Whitney U test or Independent Samples T-Test. A chi-square test was used for categorical variables. Differences in PANSS, HCR-20, M-WCST, and WMS performances were calculated between SCZ and control groups by a nonparametric Mann-Whitney U test or Independent Samples T-Test.

Correlations between serum protein factor concentrations were analyzed using either Pearson (normal distribution variable) or Spearman (non-normal distribution variable) correlations.

Spearman correlations between BDNF, TrkB, CREB and psychopatological/cognitive/memory measures were made in SCZ patients. In the patient group, with total HCR-20 score, total PANSS score, and MQ scores as dependent variables, as well as signaling factors, gender, marital status, age, BMI, smoking, and education as independent variables, multivariate linear regression analysis was performed to investigate the influential factors of risk of violence, symptoms of mental disorders, and cognitive function scores.

Multivariable logistic regression analysis was performed using stepwise logistic regression with forward selection and backward elimination by removing variables with p-value greater than 0.05. Results were expressed as odds ratio (OR) with 95% confidence interval (CI). Finally, to evaluate the markers’ ability to predict CR, analysis of the receiver operating characteristics curve (ROC) by the area under the curve (AUC) was performed according to logistic regression. We conducted binary logistic regression models to examine factors that potentially contributed to violent behavior in patients with SCZ. All statistical tests were two-tailed, and the significance level was set at 0.05.

## 3 Results

### 3.1 Demographic and psychopathological characteristics

**Table 1** shows the demographic data of the SCZ group (n=62) and the healthy control group (n=62). No statistically significant differences were observed between groups in terms of gender, smoking, age, and education (P > 0.05). However, statistically significant differences were identified in marital status family history and BMI between the two groups (P < 0.05).

**Table 1:** The demographic and clinical characteristics between SCZ patients and controls.

Characteristic	SCZ(n=62)	Control(n=62)	t/Z/χ²	P
Gender (male/female)^a^	32/30	31/31	0	1
smoking (yes/no)^a^	16/46	16/46	0	1
Marital status (single/married)^a^	44/18	27/35	8.4358	0.0036
Family history (yes/no)^a^	15/47	4/58	7.1599	0.00745
Age^b^	33.66 ± 12.00	35.24 ± 10.29	5.12	0.2834
BMI^c^	22.44 ± 4.10	24.80 ± 4.07	-3.2131	0.0016
Education^b^	12.10 ± 3.00	12.42 ± 3.57	6.05	0.6749
Time of illness (month)	7.403 ± 3.713			
Positive and Negative Syndrome Scale				
positive score (P_score)	21.84 ± 7.05			
negative score (N_score)	23.35 ± 4.94			
PANSS total score	96.84 ± 10.21			

^a^chi-square test.

^b^nonparametric test, Mann-Whitney U.

^c^t test.

Within the SCZ group, gender difference of PANSS severity scores were analyzed. The result showed no significant difference between the male and female patients with respect to P_score and PANSS total score (P < 0.05) (**Table 2**). However, female patients demonstrated a significantly higher N_score (P = 0.02) (**Table 2**). This finding suggested that female patients exhibited greater impairment in emotional withdrawal, poor rapport, passive/apathetic social withdrawal, lack of spontaneity/flow of conversation, and active social avoidance. These results indicate that first-episode drug- naive female SCZ patients were more likely to be dominated by negative symptoms. These data would inform subsequent analysis.

**Table 2:** PANSS severity scores for male and female patients.

Measure	Male(n=32)	Female(n=30)	t/Z	P
P_score ^a^	22.97 ± 5.46	20.63 ± 8.35	-7.00773	0.67
N_score ^b^	22.0 ± 4.59	24.8 ± 4.96	-2.3068	0.02
PANSS total score^b^	96.125 ± 8.01	97.600 ± 12.24	-0.55768	0.58

^a^nonparametric test, Mann-Whitney U

^b^t test.

### 3.2 Expression differences in BDNF, TrkB and CREB

**Figure 1:**
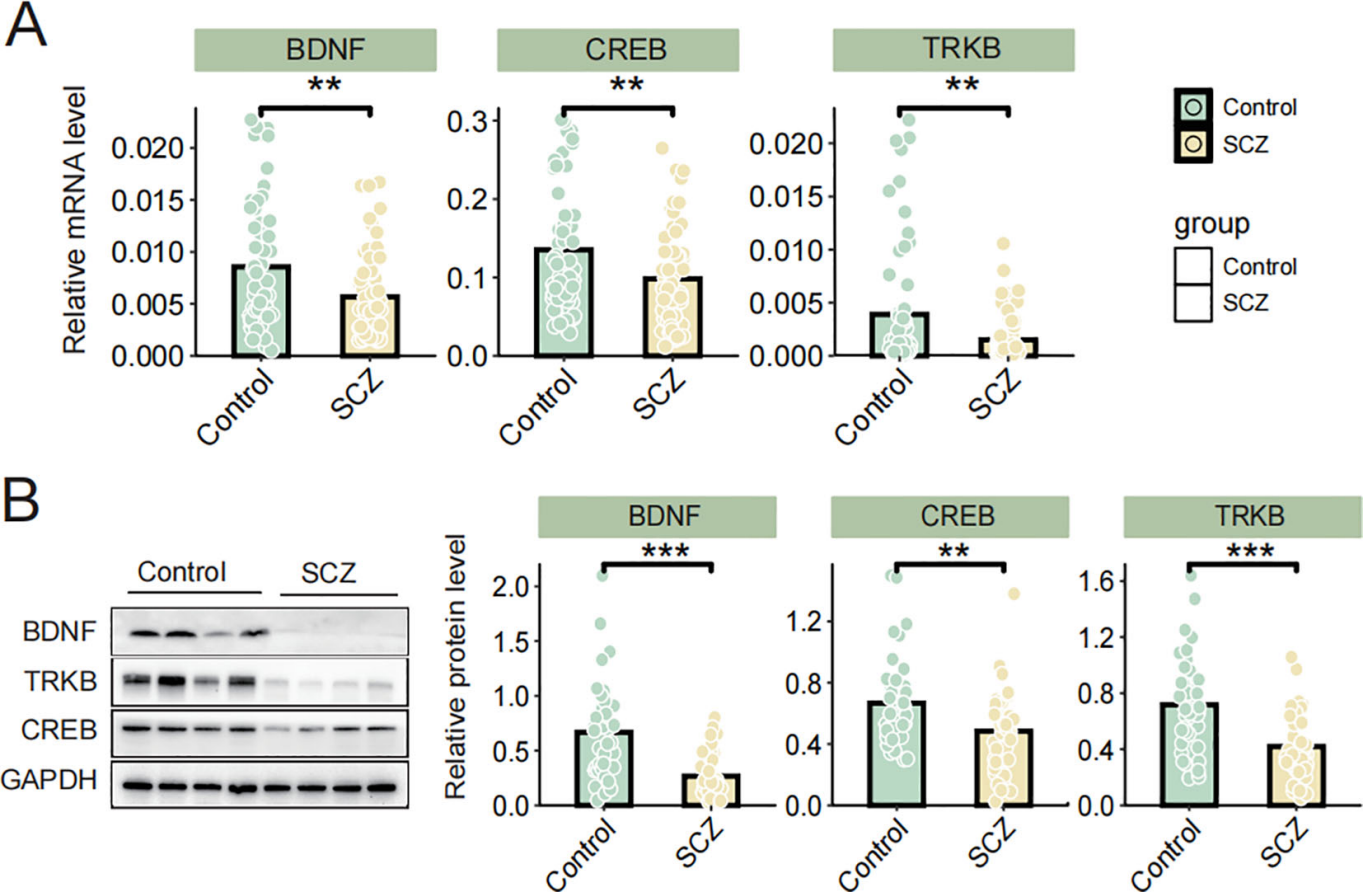
**(A)** The mRNA levels of BDNF, TrkB, and CREB were determined via qRT-PCR; **(B)** The protein levels of BDNF, TrkB and CREB were determined by western blot. Data are expressed as the mean ± SD. **P < 0.01 or ***P < 0.001 was considered as significant.

Given the crucial role of the BDNF/TrkB/CREB signaling pathway in SCZ, we analyzed the expression differences in BDNF, TrkB, and CREB at both the mRNA and protein levels in peripheral blood lymphocytes between the SCZ group and healthy controls. The qPCR results revealed significant reduced BDNF, TrkB and CREB mRNA levels in individuals with SCZ compared to healthy controls (**Figure 1A**). Consistently, immunoblotting results demonstrated a downregulated expression of BDNF, TrkB, and CREB in SCZ groups (**Figure 1B**). Additionally, the correlations among BDNF, TrkB and CREB protein level were analyzed using Pearson correlation analyses. In both the SCZ and control groups, BDNF was positively correlated with CREB and TrkB levels (P< 0.05). Moreover, there was a positively correlation between TrkB and CREB (P< 0.05) (**Table 3**). These finding revealed significant associations among BDNF, TrkB, and CREB in SCZ patients. In summary, the reduced expression of BDNF, TrkB, CREB in SCZ, along with their correlation, highlighted the disrupted neurotrophic signaling. This disruption may underlie the pathophysiology of this disorder.

**Table 3:** Pearson correlation analysis of BDNF, TrkB, and CREB protein levels.

Variable pair	SCZ (n=40)		Control (n=40)	
	r	P	r	P
BDNF& TrkB	0.607	<0.001	0.706	<0.001
BDNF&CREB	0.397	0.011	0.433	0.005
TrkB&CREB	0.334	0.035	0.631	<0.001

### 3.3 Comparison of risk of violence between SCZs and controls

**Figure 2:**
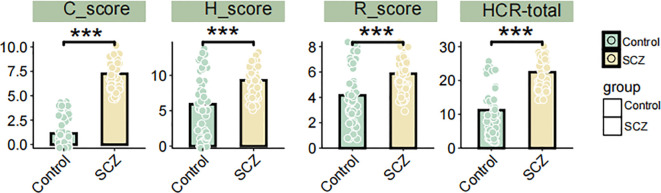
Differences in HCR-20 total, H, C, and R subscale scores between SCZ patients and controls. *** *P* < 0.001, Student’s t-test.

We compared the differences in HCR-20 scores and HCR-20 subscale scores between the SCZ group and the control group. The result demonstrated that the total HCR-20 score, as well as H, C, R subscale scores were significantly higher in SCZ patients compared to the controls (P< 0.001) (**Figure 2A**). **Table 4** shows the descriptive statistics distribution of HCR-20 total and subscale scores. The mean item score on the total HCR-20 scale, an assessment of violence risk, were 22.44 (SD 4.27) in SCZ patients and 11.23 (SD 7.36) in health controls (**Table 4**). In the SCZ patients, the medium, min and max item of total HCR-20 score were 23.5, 14, 30, respectively, and those for control group were 9.5, 2, and 26 (**Table 4**). Moreover, the mean item score on the H scale was 9.31 (SD 1.97) for SCZ (n = 62) and was 5.92 (SD 4.19) for control (n = 62) (**Table 4**). Regarding the C and R scales, the mean values in the SCZ group were 7.26(SD 1.42) and 5.87(SD 1.41), respectively, which were higher than those of the control group (**Table 4**). Likewise, the medium values of C and R scales in the SCZ group were 7 and 6, respectively, surpassing those of the control group (**Table 4**). Collectively, these findings illuminated the overall distribution of HCR-20 scores and their subscale scores. The evaluated total and subscale scores in SCZ group suggest a higher risk of violent behavior.

**Table 4:** Descriptive statistics distribution of HCR-20 total score and subscale scores.

Measure	SCZ (n=62)				Control (n=62)					
	mean	SD	medium	min	max	mean	SD	medium	min	max
HCR-20 H	9.31	1.97	9	5	13	5.92	4.19	6	0	14
HCR-20 C	7.26	1.42	7	5	10	1.14	1.54	0	0	4
HCR-20 R	5.87	1.41	6	3	8	4.16	2.09	4	1	8
HCR-20 total	22.44	4.27	23.5	14	30	11.23	7.36	9.5	2	26

HCR-20, Historical Clinical Risk Management–20; H, Historical scale; C, Clinical scale; R, Risk Management scale; Min, minimum; Max, maximum.

### 3.4 Cognitive performance in M-WCST test

The results of the assessment of cognitive flexibility and shifting attention, detailed in **Table 5**, provide a comprehensive comparison of Modified Wisconsin Card Sorting Test (M-WCST) for both the SCZ and control groups. The table includes mean scores, standard deviations, F scores, and p-values for key cognitive metrics. The comparative analysis encompassed various cognitive measure indices, including correct responses, the number of trials required to complete the first category, the number of completed categories, total errors, perseverative errors, and non-perseverative errors.

**Table 5:** Modified Wisconsin Card Sorting Test (M-WCST) performance in SCZ and control groups.

M-WCST index	SCZ	Control	Z	P
Correct response (CR)	18.87 ± 6.57	28.64 ± 3.13	4.38	< 0.001
Number of trials to complete the first category (TCFC)	14.27 ± 13.51	12.14 ± 4.02	6.28	0.8078
Number of completed categories (CC)	1.35 ± 1.28	3.63 ± 0.99	4.44	< 0.001
Total errors (TE)	29.13 ±:6.57	19.35 ± 3.13	17.64	< 0.001
Preservative errors (PE)	13.52 ± 5.66	10.60 ± 2.66	11.35	< 0.0016
Non-preservative errors (NPE)	15.61 ± 6.44	8.76± 2.65	15.59	< 0.001
Time for task completion (s)	267.77 ± 161.55	120.84 ± 25.35	17.92	< 0.001

Nonparametric test, Mann-Whitney U.

Data are expressed as the mean ± SD.

In the SCZ group, total errors (p< 0.001), perseverative errors (p < 0.001), non-perseverative errors (p < 0.001), and the total time required to complete the test (p < 0.001) were significantly higher than those in the control group. Conversely, the number of correct responses (p < 0.001) and the number of completed categories (p < 0.001) in the SCZ group were significantly lower compared to the control. There were no significant differences in number of trials to complete the first category between the two groups (p > 0. 05).

These findings suggested that individuals with SCZ may exhibit notable deficits in cognitive flexibility and executive function. The increased error rates and longer completion times, as well as their reduced ability to generate correct responses and complete categories indicated potential impairments in their cognitive processing capabilities.

### 3.5 Comparative analysis of WMS scores between SCZ patients and controls

The presented data compared the memory performance between individuals with SCZ and healthy controls. The mean and standard deviation of the SCZ and control groups on the Wechsler Memory Scale (WMS) indices and subtests are initially presented in **Table 6**, accompanied by Z values derived from the Mann-Whitney U test. The findings revealed that SCZ patients exhibited significantly poorer performance than the control group across all indices and subtest scores, with all differences reaching statistical significance (p < 0.001).

**Table 6:** Wechsler Memory Scale-Revised scores of the participants.

Index	Subtests	SCZ	Control	Z	P
Memory quotient (MQ)		60.92 ± 34.88	105.39 ± 10.69	4.94	< 0.001
Long-term Memory	1-100	5.87 ± 3.97	9.79 ± 2.34	1.29	< 0.001
	100-1	8.34 ± 3.67	12.21 ± 2.35	2.75	< 0.001
	accumulation	7.02 ±:3.00	9.69 ± 1.65	0.66	< 0.001
Short-term Memory	Picture memory	5.77 ± 2.54	7.69 ± 1.92	0.71	< 0.001
	Picture recognition	3.21 ± 3.14	8.35 ± 3.82	2.64	< 0.001
Immediate Memory	recitation of numbers	12.66 ± 4.93	15.77 ± 1.25	2.17	< 0.001

Nonparametric test, Mann-Whitney U test. 1-100: counting from 1 to 100. 100-1: counting down from 100 to 1.

Accumulation: initiate at 1, increment by 3 in each step, and terminate upon reaching or exceeding 49.

The MQ, derived from the weighted total of subtest scores, served as an indicator of an individual’s memory capability. A particularly striking finding was observed in MQ score. The SCZ group had a mean of 60.92 with a standard deviation of 34.88. In contrast, the healthy control group achieved a mean MQ score of 105.39 with a standard deviation of 10.69. This substantial difference underscored a significant disparity in memory performance between the two groups (p < 0.001, **Table 6**).

These results underscored profound memory impairments experienced by individuals with SCZ, revealing the extent to which their memory performance deviated from that of individuals without the disorder. The significant differences across all measured indices and subtests reflect the pervasive impact of SCZ on cognitive functions, particularly memory-related tasks.

### 3.6 Correlations between BDNF, TrkB, CREB and psychopatological/cognitive measures

The relationship between BDNF, TrkB, and CREB with various indices related to psychopathology and cognition function in patients with SCZ remains undefine. To address this, we first examined the correlations among these three signaling factors and several key assessment scores, including total PANSS score, total HCR-20 score and their subscale scores.

**Figure 3:**
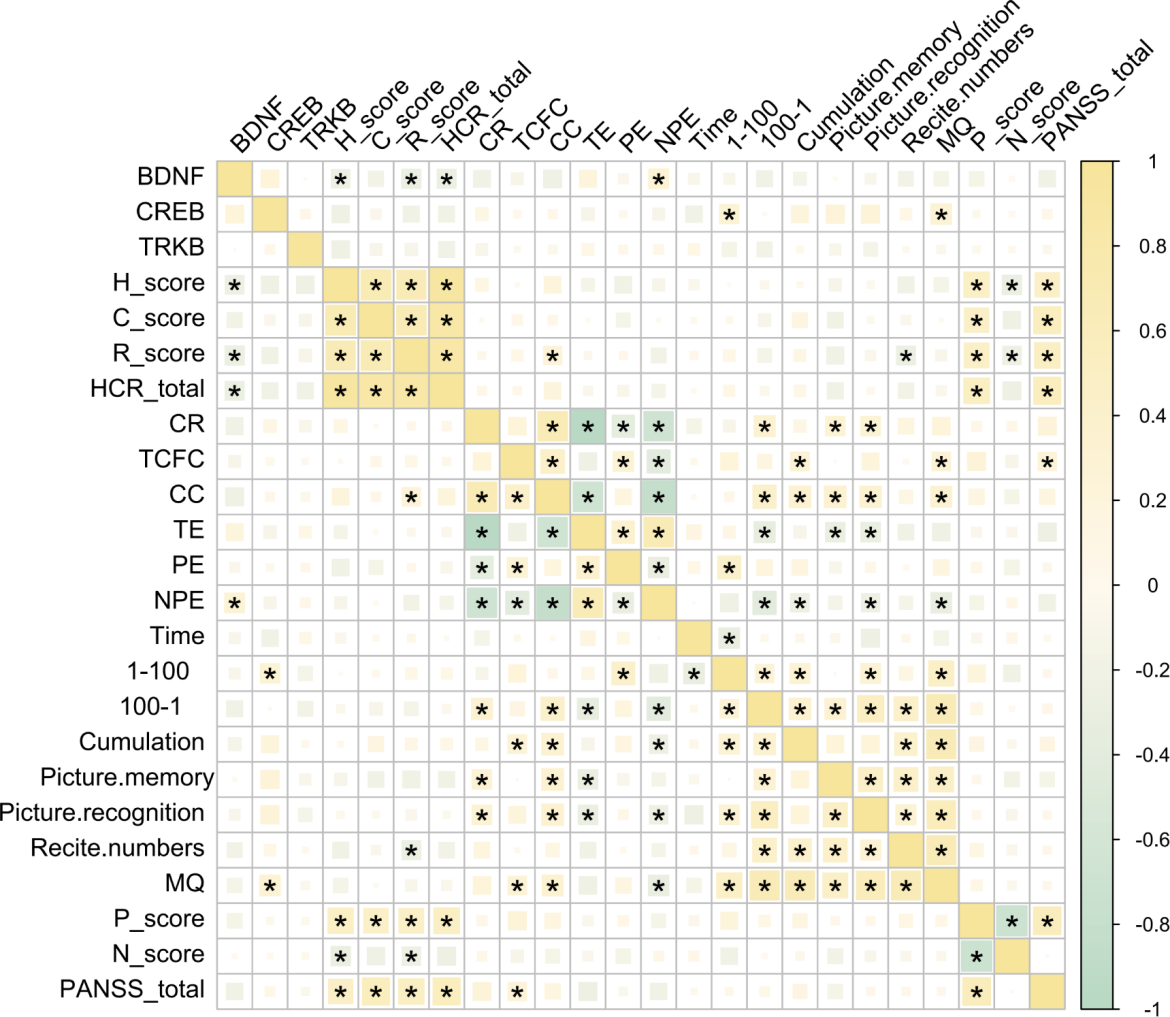
Correlations among BDNF, TrkB, CREB and PANSS, HCR-20, M-WCST WMS indices. *P < 0.05 was considered as significant.

Our findings revealed no significant correlations between BDNF, TrkB, or CREB and the total PANSS score, suggesting that these signaling factors may not directly influence the overall symptom severity of schizophrenia as measured by PANSS. However, a significant negative correlation was found between BDNF and the H-score, R-score, and the total HCR-20 score. This finding suggests that lower levels of BDNF were associated with higher risk factors and historical indicators of violence and risk in these patients (**Figure 3**). Interestingly, BDNF was positively correlated with NPE in patients with SCZ. This finding suggested that higher levels of BDNF may be associated with increased non-perseverative errors, indicating a potential link to difficulties in attention and decision-making processes (**Figure 3**).

CREB, on the other hand, appeared to influence memory functions in individuals with SCZ, which was evidenced by its significant positive correlations with both (MQ and the 1–100 scores (**Figure 3**). This suggested that higher CREB levels may be associated with better memory performance in this population. Overall, these results indicated distinct roles for BDNF and CREB in the cognitive and psychopathological profiles of schizophrenia, with BDNF more closely linked to executive function and CREB more associated with memory capabilities. This differentiation may provide valuable insights for future research aimed at understanding the underlying mechanisms of cognitive deficits in SCZ.

Further analysis of pairwise correlation analysis among the HCR-20, M-WCST, WMS, and PANSS scales and their respective subscales provide additional insights. The HCR-20 total score and its subscales showed significant positive correlations with the P-score and the total PANSS score, indicating significant association with violent risk and more severe psychopathological symptoms (**Figure 3**). Conversely, the N-score exhibited a significant negative correlation with the H and R subscales, suggesting that higher scores in these areas may be linked to lower negative symptoms.

Moreover, CR and CC were significantly positively correlated with long-term memory (measured by the 100–1 task) and short-term memory (as assessed through figural memory and picture recognition tasks). This suggested the correlation between better performance in these cognitive tasks with improved memory function. Additionally, the MQ demonstrated a significant positive correlation with both TCFC and CC, further supporting the link between cognitive flexibility and memory performance (**Figure 3**). Importantly, we did not find any significant correlations between the PANSS subscales and the WMS indices (**Figure 3**).

In summary, these findings highlight the distinct roles of BDNF, TrkB, and CREB in various psychopathological, cognitive, and memory measures. They could provide valuable insights into the neurobiological mechanisms underlying various mental health disorders and highlight potential therapeutic targets for enhancing cognitive function and memory resilience.

### 3.7 Influential factor and predictor related to violence and cognitive function

To investigate the relationships between various biological and clinical factors and their influence on violent behavior and cognitive function in first-episode drug-naive SCZ patients, we conducted a multivariate linear regression analysis using the total HCR-20 score and the MQ score as dependent variables. Independent variables included several factors: BDNF, TrkB, CREB, gender, time of illness, marital status, age, BMI, smoking status, family history of mental illness, education level, P-score, N-score, and total PANSS score. The analysis identified CREB(estimate< 0), N-score (estimate< 0), and total PANSS score(estimate>0) as significant predictors of the total HCR-20 score (P < 0.05) (**Table 7**). This suggests that higher levels of CREB and N-score were associated with a decreased risk of violent behavior, whereas a higher total PANSS score, reflecting more severe psychopathological symptoms, correlated with an increased risk of violence in patients with SCZ. Furthermore, CREB emerged as a significant predictor of the MQ score (P < 0.05), highlighting its potential role in cognitive functioning (**Table 8**). Those suggest that CREB may play a crucial role in both violent behavior and cognitive function in patients with SCZ.

**Table 7:** Multivariate linear regression analysis of total HCR-20 score and demographic index in patients- stepwise regression method.

Term	Estimate	std.error	t value	p.value	95%CI	
					LL	UL
(Intercept)	0.67	0.05	12.66	<0.0001	0.57	0.78
CREB	-0.15	0.06	-2.45	0.02	-0.28	-0.03
education	0.08	0.06	1.49	0.14	-0.03	0.19
BMI	0.09	0.05	1.74	0.09	-0.01	0.19
P_score	-0.26	0.17	-1.54	0.13	-0.60	0.08
N_score	-0.33	0.12	-2.62	0.01	-0.57	-0.08
PANSS_total	0.47	0.12	3.74	<0.0001	0.22	0.72

**Table 8:** Multivariate linear regression analysis of MQ score and demographic index in patient-stepwise regression method.

Term	Estimate	std.error	Statistic	p.value	95%CI	
					LL	UL
(Intercept)	-0.61	0.13	-4.53	<0.0001	-0.88	-0.34
CREB	0.43	0.15	2.87	0.01	0.13	0.73
BDNF	-0.23	0.17	-1.40	0.17	-0.56	0.10

To further explore the predictors of violence in SCZ, we employed multivariate logistic regression analysis. According to the Meyer et al. ’s report (27), patients with a total HCR-20 score of ≥ 21 were categorized as the violent patient group, and those with a total HCR-20 score of < 21 were assigned to the nonviolent patient group. During the analysis, to address the multicollinearity among variables, principal component analysis (PCA) was used to combine the three highly correlated variables P_score, N_score and PANSS_total into a single principal component, PC1. Among all variables analyzed, only PC1 showed a statistically significant positive association with the likelihood of violent behavior in individuals with SCZ (P < 0.05, OR = 2.32). In contrast, other variables such as gender, age, and BMI did not demonstrate significant effects (**Table 9**). These findings suggest that the composite information reflected by P_score, N_score and PANSS_total might be a critical factor influencing violent behavior in this population.

**Table 9:** Association between the violence and BDNF, TrkB, CREB, and demographic index using multivariate logistic regression analysis.

Term	Coef	se(coef)	Chisq	p.value	OR	95%CI	
						LL	UL
(Intercept)	-0.22	0.86	0.06	0.81	0.80	-2.10	1.60
BDNF	0.28	0.42	0.39	0.53	1.33	-0.64	1.18
CREB	0.64	0.39	2.58	0.11	1.89	-0.13	1.61
TRKB	0.70	0.64	1.14	0.29	2.01	-0.55	2.24
Time of illness	-0.31	0.42	0.47	0.49	0.73	-1.24	0.59
gender	-0.21	0.77	0.06	0.80	0.81	-2.04	1.46
smoking	-0.57	0.81	0.45	0.50	0.56	-2.36	1.11
age	-0.15	0.38	0.14	0.71	0.86	-0.99	0.64
education	-0.47	0.35	1.65	0.20	0.63	-1.25	0.24
BMI	0.16	0.36	0.17	0.68	1.17	-0.58	0.96
marital status	0.78	0.72	1.05	0.31	2.17	-0.68	2.45
family history	0.24	0.79	0.08	0.78	1.27	-1.36	2.07
PCl	0.84	0.26	13.06	<0.001	2.32	0.36	1.49

OR, Odds Ratio; CI, confidence interval; LL, lower limit; UL, upper limit; After principal component analysis, PC1 was used to replace these three original variables (P_score、N_score and PANSS_total) for modeling.

### 3.8 Multivariable logistic regression and ROC curve analysis of the three signaling factors for patients with SCZ

**Figure 4:**
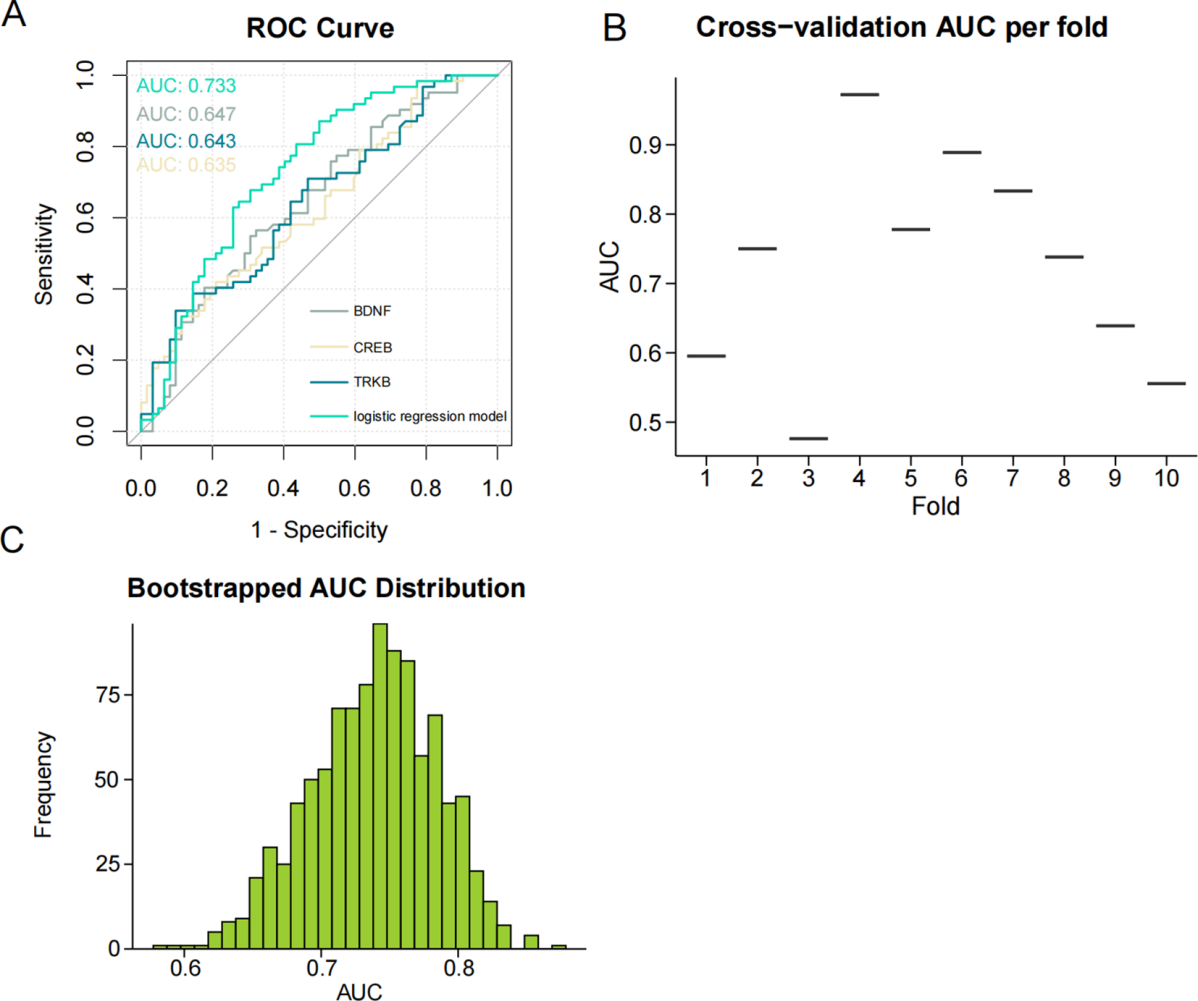
**(A)** ROC curve for the three signaling factors and logistic regression model in predicting SCZ (AUC = 0.733). ROC, receiver operating characteristic; AUC, area under the ROC curve. **(B)** The result of Cross-validation AUC. **(C)** The AUC distribution result of bootstrapping method.

**Figure 5:**
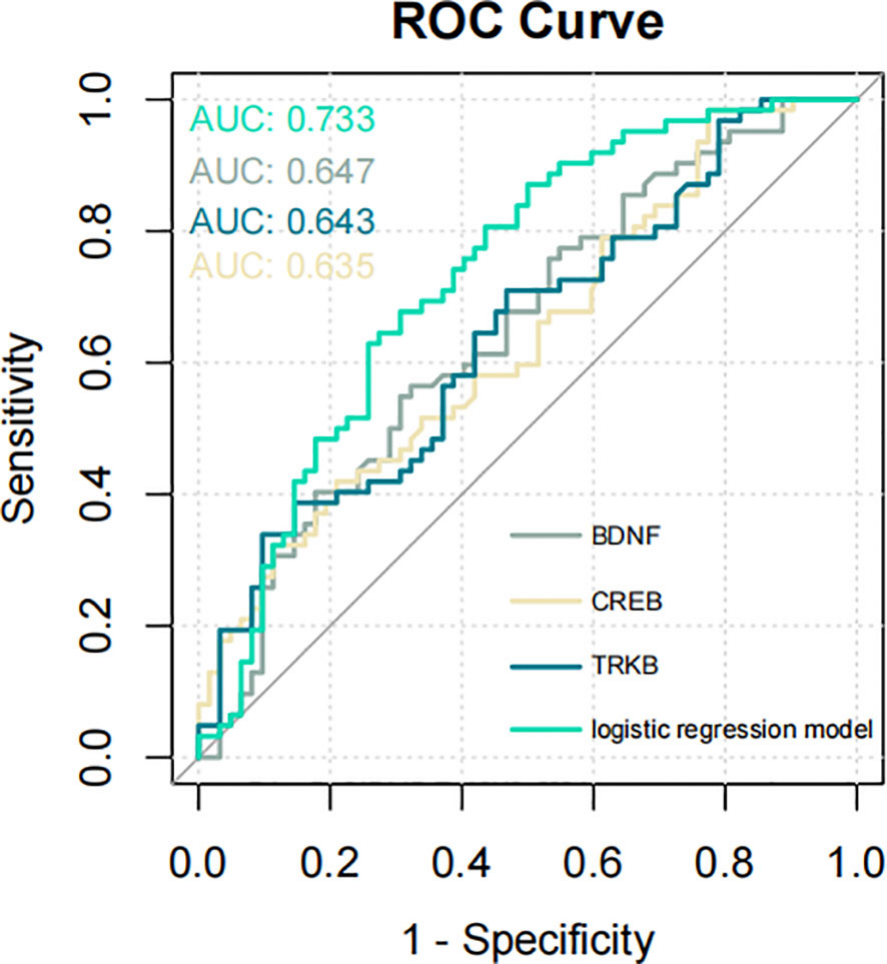
ROC curve illustrating the predictive performance of the three signaling factors and the logistic regression model for SCZ (AUC = 0.733). ROC, receiver operating characteristic; AUC, area under the ROC curve.

To identify the independent variables that significantly influenced the risk of developing SCZ, we further examined the relationship between BDNF, TrkB, CREB, and SCZ. We found that BDNF, TrkB, and CREB were independently associated with SCZ (OR *<* 1, *P <* 0.05), demonstrating a statistically significant association with the disorder (**Table 10**). To evaluate the predictive validity of these factors in relation to SCZ, we conducted a ROC curve analysis. The results revealed that the AUC for each individual predictor was as follows: 0.647 for BDNF, 0.643 for TrkB, and 0.635 for CREB. These values suggested that each biomarker possess moderated the diagnostic accuracy in predicting the possibility of SCZ (**Figure 4A**). Moreover, when incorporating BDNF, TrkB, and CREB into a multivariate logistic regression model, the AUC increased to 0.733. **Figure 4B** shows the AUC for each fold in the cross-validation process, which was conducted to assess the stability and generalizability of our model. A total of 10 folds were used, yielding an average AUC of 0.7226 across all folds. These results suggest that the model possessed reasonable discriminatory power. Additionally, in the histogram from the bootstrapping analysis, the AUC distribution is centered around 0.870, with the spread reflecting the variability in the performance of the model (**Figure 4C**). This indicated that the combination of the three independent variables worked better than applying any variable alone. The enhanced AUC underscored the importance of a multifactorial approach in assessing the risk of SCZ, allowing a comprehensive evaluation of the interplay between biological markers and the disorder (**Figure 5**). Our findings emphasized the significance of BDNF, TrkB, and CREB as independent factors associated with SCZ. ROC curve analysis highlighted their individual predictive capabilities and illustrated the advantages of a multivariate model in improving predictive accuracy. These insights could guide future research and clinical practices aimed at early identification and intervention for individuals at risk of developing SCZ.

**Table 10:** Association between the SCZ and BDNF、TrkB、CREB using multivariate logistic regression analysis.

Term	Estimate	Std. Error	z value	Pr(>|z|)	OR	95%CI	
						LL	UL
(Intercept)	-0.07	0.20	-0.34	0.73	0.93	0.62	1.38
BDNF	-0.60	0.22	-2.71	0.0067	0.55	0.34	0.83
CREB	-0.44	0.21	-2.11	0.035	0.64	0.42	0.96
TRKB	-0.67	0.28	-2.40	0.016	0.51	0.27	0.83

## 4 Discussion

SCZ is a complex psychiatric disorder characterized by cognitive impairments and a potential risk of violent behavior, presenting significant challenges for both individuals and society. Recent studies by Guo et al. have indicated that BDNF, TrkB, and CREB play crucial roles in mental health (11). These molecules are not only involved in the growth and survival of neurons, but also closely play a role in emotional regulation and cognitive function. In this study, we assessed the expression levels of BDNF, TrkB, and CREB in peripheral blood lymphocytes of first-episode drug- naive patients with SCZ. We hope to reveal the potential role of these biomarkers in SCZ and provide new insights into early intervention and treatment strategies. We found that both the mRNA and protein levels of BDNF, TrkB, and CREB were significantly downregulated in SCZ patients than those in healthy controls. This reduction was accompanied by significant differences in HCR-20, WMS, and M-WCST-related scores. Furthermore, the three factors were associated with scores on the PANSS, the HCR-20, as well as WMS and WCST. These findings provide robust support for the role of BDNF, TrkB, and CREB in the pathophysiology of SCZ, offering a new lens through which to understand their impact on cognitive impairments and violent tendencies. Multivariate linear regression and logistic regression analyses further revealed key factors influencing cognitive function and violence risk, providing a scientific basis for early intervention and treatment strategies for SCZ.

In our study, we observed significant differences in demographic characteristics, such as marital status, family history, and BMI, between the SCZ and control groups. It is worth noting that PANSS total score did not differ significantly between male and female patients, though differences were significant for negative symptoms. This finding aligns partially with the study of Patrick et al. (28), which reported no significant gender in positive symptoms or the positive symptom total score. In our study, the female first-episode, drug-naive patients mainly characterized by negative symptoms such as emotional flatness, social withdrawal and lack of motivation. However, while Patrick et al. found that male patients exhibited more severe negative symptoms (P < 0.05), our results did not support this observation. We hypothesize that these discrepancies may stem from differences in sample characteristics. Patrick et al.’s study involved geriatric chronic psychiatric inpatients and continuously institutionalized for at least 10 years, while our study focused on patients with shorter durations of illness. Chronic institutionalization, with its associated factors such as social isolation, lack of stimulation, and limited opportunities for social interaction, could exacerbate negative symptoms, potentially explaining the observed severity in male patients in their study. Other research further supports the variability in gender differences depending on illness duration and treatment history. For example, one study reported no significant difference in PANSS Positive scores between men and women, but male scored higher on PANSS negative and general scales than females (29). It may also be due to the illness duration of the included patients. Conversely, another investigation (30) reported that in first-episode drug-naive SCZ male patients scores higher than females on the PANSS total, positive psychopathology subscale scores, but not on the negative subscale score. These findings suggest that gender differences in SCZ symptomatology may vary depend on the stage of the illness and treatment history. The discrepancy in results between studies could be attributed to differences in patient populations, such as the stage of the disorder (first-episode versus chronic) and whether patients have been previously treated with antipsychotic medications. In first-episode, drug-naive patients, the absence of significant differences in negative symptoms between genders might indicate that such symptoms become more pronounced with prolonged illness and treatment. This underscores the importance of considering the illness stage and treatment status when evaluating gender differences in schizophrenia symptoms. Future research should focus on longitudinal assessments to better understand how these factors interact over the course of the illness to influence symptom profiles in male and female patients.

BDNF plays a vital role in neurogenesis, synaptic plasticity, cognition, and neurotransmission (31), with the BDNF/TrkB/CREB signaling pathway being critical for ameliorating neuronal injury and cognitive impairment (11). In the present research, we observed significantly reduced levels of BDNF, TrkB, and CREB in SCZ patients, both at the mRNA and protein levels, aligning with findings from previous studies. Studies have demonstrated that animal models of SCZ typically exhibit low levels of BDNF (32). Additionally, decreased mRNA levels of BDNF and its receptor, TrkB, have been detected in plasma in SCZ patients (33). CREB, an upstream regulator of BDNF, was also shown decreased expression in SCZ patients (34). Furthermore, several studies have linked low level of BDNF with depressive mood and cognitive dysfunction (35–38). These findings suggest the significant correlation between these signaling factors and SCZ, indicating their potential as key molecular targets for understanding the pathogenesis and progression of SCZ. Targeting the BDNF/TrkB/CREB pathway offers promising therapeutic strategies for alleviating symptoms and improving cognitive and emotional outcomes in SCZ patients. This evidence highlights the pathway’s role as a critical area for future research and potential clinical applications.

Subsequently, we compared the differences in violence and cognition between the SCZ and control groups. Risk estimates based on the HCR-20 scores indicated that the total H, C, and R subscale scores were significantly higher in the SCZ group, suggesting a markedly increased risk of violence compared to the control group. The WCST analysis revealed that the SCZ group exhibited significantly higher levels in total errors (p < 0.001), perseverative errors (p < 0.001), non-perseverative errors (p < 0.001), and total time required to complete the test (p < 0.001). The findings of WMS revealed that SCZ patients exhibited significantly poorer performance than the control group in long-term memory, short-term memory, immediate memory, as well as MQ. These findings underscore the profound cognitive deficits and heightened risk of violence in individuals with SCZ. The high violent risk observed in SCZ patients aligns with the study of Zhou et al., who conducted a meta-analysis of 3941 SCZ inpatients in China. They found that an average of 35.4% of patients exhibited aggressive behavior, with rates as high as 53.2% in some cases (39). These findings emphasize the urgent need for targeted interventions to manage violence risk in this population.

The WCST results are consistent with prior research by James et al., who found that patients with SCZ performed poorly on multiple cognitive tasks, including completing fewer categories successfully, making more perseverative errors and responses, requiring more trials to succeed at the first category, and having significantly lower conceptual level responses compared to controls (40). This cognitive profile suggests that therapeutic strategies focusing on cognitive rehabilitation could be beneficial. Moreover, the consistency between our findings with those reported by James et al. reinforces the validity of our results, emphasizing the importance of ongoing research in understanding and addressing the complex challenges faced by individuals with SCZ. These findings also support previous studies indicating that cognitive impairment in both a diagnostic criterion and treatment target for SCZ (41).

We further comprehensively analyzed the effects of three signaling factors, along with demographic and psychopathological characteristics on the risk of violence and cognitive function in patients with a first-episode of SCZ. The result showed that the *CREB*, N_score, and PANSS_total score significantly influenced the total HCR-20 score, with CREB being crucial in determining the MQ. Further investigation revealed that PC1 score was an independent factor associated with the occurrence of violence in first-episode drug-naive SCZ patients. These results underscore the critical role of *CREB* in both the propensity for violence and cognitive function among SCZ patients. They also highlight the importance of CREB in violence and cognitive function of SCZ patients. Animal studies have reported the association between phosphorylation of the CREB, CREB1 polymorphisms and aggression or major depressive disorder (MDD) (42). In humans, the *CREB1* gene is located on chromosome 2, and it encodes the cyclic adenosine phosphate reactive element binding protein (CREB), which is broadly expressed in the human brain and active only when phosphorylated (43). CREB contributes to neuronal signal pathway and emotional reactivity (44, 45). In an investigation of male Chinese adolescents, Zhang et al. reported that CREB1 rs4675690 T allele was significantly associated with an increased risk of aggression (46). These results demonstrate the potential role of CREB in violence risk. Moreover, the involvement of CREB in both aggression and cognitive processes suggests that it could serve as a valuable biomarker for assessing the risk of violence and cognitive impairment in SCZ patients. Given its central role in neuronal signaling pathways and emotional regulation, CREB’s modulation might offer new therapeutic avenues.

Our study identified PANSS as a factor associated with violence, aligning with previous studies. Several studies have explored the relationship between the PANSS and violent behavior in the context of SCZ. Yi et al. found that the PANSS positive subscale significantly contributed to the development of violent behavior in SCZ patients (47), consistent with our study. This association may be attributed to hallucinations caused by positive symptoms, which can provoke the patient’s unpleasant feelings (anger, sadness). An investigation also highlights that total PANSS scores and Positive subscales are associated with increased violence risk (48). Moreover, a meta-analysis of 110 studies on risk factors for violence in psychosis concludes that higher general symptom ratings and higher total PANSS ratings are linked with violence (49). With regards to specific positive symptoms, violence is associated with higher excitement ratings and higher positive symptoms ratings (50). According to their findings, the violence risk of SCZ may increase with increasing total PANSS score and P scores.

Our study had several limitations that should be acknowledged. Firstly, the relatively small sample size (62 patients and 62 healthy controls) may limit the generalizability of the findings to broader populations. Secondly, the logistic regression model used in this study may have inherent limitations such as potential biases in variable selection and model assumptions, affecting its predictive accuracy. Third, as a cross-sectional study, our research only identifies associations and cannot establish causality between these observation factors and violence. Additionally, the samples were drawn from a single center (the Affiliated Mental Health Center of Kunming Medical University), potentially limiting the applicability of the results to other regions or populations. In addition, despite efforts to control for key clinical variables, residual confounding from unmeasured factors, such as environmental influences, trauma history, or substance use, cannot be entirely excluded. Future research involving larger sample sizes, longitudinal designs, and multi-center studies would enhance further validate and deepen the study’s conclusions. We also highlight the need for further investigate the role of CREB in patients with SCZ. Longitudinal studies could observe changes in these factors over time and their impact on treatment response. Such research would deepen insights into the mechanisms underlying SCZ and help tailor more effective interventions.

In conclusion, this study found that patients with SCZ exhibited significantly downregulated levels of BDNF, TrkB, and CREB compared to healthy controls. These reductions were associated with distinct sociodemographic, psychopathological, and cognitive differences between the two groups. Specifically, lower levels of these signaling factors were correlated with higher risk of violent behavior and poorer cognitive performance in in first-episode drug-naive SCZ patients. Additionally, BDNF, TrkB, and CREB emerged as important indices for predicting SCZ. These results highlight the importance of integrating neurobiological factors with clinical assessments to better manage violence risk and cognitive deficits in SCZ. Targeting CREB expression, in particular, may offer a novel therapeutic avenue, paving the way for more effective interventions to improve outcomes for SCZ patients.

## Data Availability

The data presented in the study are available in Figshare, doi 10.6084/m9.figshare.29117450.

## References

[1] Ayilara GO Owoyele BV. Neuroinflammation and microglial expression in brains of social-isolation rearing model of schizophrenia. IBRO Neurosci reports. (2023) 15:31–41. doi: 10.1016/j.ibneur.2023.05.010 PMC10285239 37359498

[2] Sameer J Mandy J Peter M. Schizophrenia. Lancet. (2022) 399:473–86. doi: 10.1016/S0140-6736(21)01730-X 35093231

[3] Zixiang W Qinqin L Yinghua Z Xiaoni G Meihong X Xiangyang Z. Superoxide dismutase, BDNF, and cognitive improvement in drug-naive first-episode patients with schizophrenia: A 12-week longitudinal study. Int J Neuropsychopharmacol. (2021) 25:128–35. doi: 10.1093/ijnp/pyab065 PMC8832226 34622272

[4] Yang YJ Li YK Wang W Wan JG Yu B Wang MZ. Small-molecule TrkB agonist 7,8-dihydroxyflavone reverses cognitive and synaptic plasticity deficits in a rat model of schizophrenia. Pharmacology Biochem Behavior. (2014) 122:30–36. doi: 10.1016/j.pbb.2014.03.013 24662915

[5] Green MF Kern RS Braff DL Mintz J. Neurocognitive deficits and functional outcome in schizophrenia: are we measuring the “Right stuff”? Schizophr Bulletin. (2000) 26:119–36. doi: 10.1093/oxfordjournals.schbul.a033430 10755673

[6] Fazel S Wolf A Palm C Lichtenstein P. Violent crime, suicide, and premature mortality in patients with schizophrenia and related disorders: a 38-year total population study in Sweden. Lancet Psychiatry. (2014) 1:44–54. doi: 10.1016/S2215-0366(14)70223-8 PMC4124855 25110636

[7] Weilong G Yu G Jiansong Z Xiaoping W Qiaoling S. Characteristics and associated factors of violence in male patients with schizophrenia in China. Front Psychiatry. (2023) 14. doi: 10.3389/fpsyt.2023.1106950 PMC10036402 36970285

[8] Fazel S Gulati G Linsell L Geddes JR Grann M. Schizophrenia and violence: systematic review and meta-analysis. PloS Med. (2009) 6:e1000120. doi: 10.1371/journal.pmed.1000120 19668362 PMC2718581

[9] Huang Z Kang M Li G Xiong P Chen H Kang L. Predictive effect of Bayes discrimination in the level of serum protein factors and cognitive dysfunction in schizophrenia. J Psychiatr Res. (2022) 151:539–45. doi: 10.1016/j.jpsychires.2022.05.004 35636029

[10] Yelmo-Cruz S Morera-Fumero AL Lakhwani S Abreu-González P. Brain-Derived Neurotrophic Factor (BDNF) and First-Episode Psychosis. A longitudinal one-year prognosis study. Actas Esp Psiquiatr. (2023) 51:130–40. doi: 10.1016/j.ajp.2020.102370 PMC10803868 37489557

[11] Guo C Liu Y Fang MS Li Y Li W Mahaman YAR. ω-3PUFAs improve cognitive impairments through ser133 phosphorylation of CREB upregulating BDNF/TrkB signal in schizophrenia. Neurotherapeutics. (2020) 17:1271–86. doi: 10.1007/s13311-020-00859-w PMC7609637 32367475

[12] Machaalani R Chen H. Brain derived neurotrophic factor (BDNF), its tyrosine kinase receptor B (TrkB) and nicotine. Neurotoxicology. (2018) 65:186–95. doi: 10.1016/j.neuro.2018.02.014 29499216

[13] Sgritta M Vignoli B Pimpinella D Griguoli M Santi S Bialowas A. Impaired synaptic plasticity in an animal model of autism exhibiting early hippocampal GABAergic-BDNF/TrkB signaling alterations. iScience. (2023) 26:105728. doi: 10.1016/j.isci.2022.105728 36582822 PMC9793278

[14] Lee B Shin E Song I Chang B. Depression in adolescence and brain-derived neurotrophic factor. Front Mol Neurosci. (2022) 15:947192. doi: 10.3389/fnmol.2022.947192 35875661 PMC9302599

[15] Zou Y Zhang Y Tu M Ye Y Li M Ran R. Brain-derived neurotrophic factor levels across psychiatric disorders: A systemic review and network meta-analysis. Prog Neuropsychopharmacol Biol Psychiatry. (2024) 131:110954. doi: 10.1016/j.pnpbp.2024.110954 38286331

[16] Shen Z Zhu J Yuan Y Ren L Qian M Lin M. The roles of brain-derived neurotrophic factor (BDNF) and glial cell line-derived neurotrophic factor (GDNF) in predicting treatment remission in a Chinese Han population with generalized anxiety disorder. Psychiatry Res. (2019) 271:319–24. doi: 10.1016/j.psychres.2018.08.111 30529313

[17] Esvald EE Tuvikene J Sirp A Patil S Bramham CR Timmusk T. CREB family transcription factors are major mediators of BDNF transcriptional autoregulation in cortical neurons. J Neurosci. (2020) 40:1405–26. doi: 10.1523/JNEUROSCI.0367-19.2019 PMC7044735 31915257

[18] Mousa HH Sharawy MH Nader MA. Empagliflozin enhances neuroplasticity in rotenone-induced parkinsonism: Role of BDNF, CREB and Npas4. Life Sci. (2023) 312:121258. doi: 10.1016/j.lfs.2022.121258 36462721

[19] Wang H Xu J Lazarovici P Quirion R Zheng W. cAMP response element-binding protein (CREB): A possible signaling molecule link in the pathophysiology of schizophrenia. Front Mol Neurosci. (2018) 11:255. doi: 10.3389/fnmol.2018.00255 30214393 PMC6125665

[20] Webster CD Douglas K Eaves D Hart SD. HCR-20: Assessing the Risk for Violence (version 2). Vancouver: Mental Health, Law, and Policy Institute,Simon Fraser University (1997).

[21] Müller-Isberner R,JD Gonzalez Cabeza S. Müller-Isberner R, Jöckel D, Gonzalez Cabeza S. Die Vorhersage von Gewalttaten mit dem HCR-20 [The Prediction of Violent Offffenses with the HCR-20]. Haina: Institut für Forensische Psychiatrie Haina (1998).

[22] Douglas KS Webster CD. The Hcr-20 violence risk assessment scheme: concurrent validity in a sample of incarcerated offenders. Criminal Justice Behav. (1999) 26:3–19. doi: 10.1177/0093854899026001001

[23] Kevin SD Melissa Y Douglas PB. Comparative validity analysis of multiple measures of violence risk in a sample of criminal offenders. Criminal Justice Behavior. (2005) 32:479–510. doi: 10.1177/0093854805278411

[24] Yang M Wong SCP Coid JW. The efficacy of violence prediction: A meta-analytic comparison of nine risk assessment tools. psychol Bulletin. (2010) 136:740. doi: 10.1037/a0020473 20804235

[25] Schretlen DJ. Modified Wisconsin Card Sorting Test: M-WCST (Professional manual). Lutz, FL: Psychological Assessment Resources (2010).

[26] Gong Y Xie G Jiang D Deng J Dai Z Zhou Q. Revised Wechsler memory scale [A]. The third Member Congress of the Chinese Psychological Association and the 60th anniversary academic conference of the association. Chin psychol Soc. (1981) 3:125–7. G. X.

[27] Meyer LF Telles LEB Mecler K Soares A Alves RS Valença AM. Schizophrenia and violence: study in a general psychiatric hospital with HCR-20 and MOAS. Trends Psychiatry Psychother. (2018) 40:310–7. doi: 10.1590/2237-6089-2017-0039 30570102

[28] Patrick M Lieber DG Ashley B Leonard W Michael P Philip DH. Gender differences in poor outcome patients with lifelong schizophrenia. Schizophr Bulletin. (2001) 27:103–13. doi: 10.1093/oxfordjournals.schbul.a006850 11215540

[29] Chengcheng P Yujia Q Tianhang Z Fude Y Lirong Z Chuanyue W. Gender differences of neurocognitive functioning in patients with first-episode schizophrenia in China. Compr Psychiatry. (2019) 95:152132. doi: 10.1016/j.comppsych.2019.152132 31669790

[30] Xiaoe L Daomin Z Guangya Z Xiangdong D Qi J Guiqin Y. Sex difference in association of symptoms and white matter deficits in first-episode and drug-naive schizophrenia. Trans Psychiatry. (2018) 8:281. doi: 10.1038/s41398-018-0346-9 PMC6298972 30563964

[31] Goren JL. Brain-derived neurotrophic factor and schizophrenia. Ment Health clinician. (2016) 29:200–10. doi: 10.9740/mhc.2016.11.285 PMC6007539 29955483

[32] Xiaojie S Yang D Leichen Xuesong L Yong C. Effects of brain-derived neurotrophic factor (BDNF) on the Schizophrenia model of animals. J Psychiatr Res. (2022) 156:538–46. doi: 10.1016/j.jpsychires.2022.10.022 36368243

[33] Kazuhiko T Koue A Yuichiro W Tatsuyuki M Makoto T Toshiyuki S. Decreased levels of brain-derived neurotrophic factor in serum of chronic schizophrenic patients. Psychiatry Res. (2002) 110:249–57. doi: 10.1016/S0165-1781(02)00127-0 12127475

[34] Shan L Cailian L Lin K Qianqian L Hongxu C Han Z. Concentration levels of BDNF, PI3K, AKT, and CREB predict depressed mood and impulsive behavior in first-episode drug- naive schizophrenia patients. Res Square (Research Square). (2022). doi: 10.21203/rs.3.rs-2230675/v1

[35] Tom KB Sarah G Walter WVDB Nico Van B Durk F. Serum brain-derived neurotrophic factor level in relation to illness severity and episode duration in patients with major depression. J Psychiatr Res. (2012) 46:285–9. doi: 10.1016/j.jpsychires.2011.12.006 22244515

[36] Tong Z Sunny XT Xiaoxi G Juan L Ran H Hai-Zhi C. Association of serum brain-derived neurotrophic factor level and early response to antipsychotic drug in first-episode patients with schizophrenia. Int J Methods Psychiatr Res. (2023) 33:e1982. doi: 10.1002/mpr.1982 37485797 PMC10804348

[37] Liu T Li H Conley YP Primack BA Wang J Li C. The brain-derived neurotrophic factor functional polymorphism and hand grip strength impact the association between brain-derived neurotrophic factor levels and cognition in older adults in the United States. Biol Res Nurs. (2022) 24:226–34. doi: 10.1177/10998004211065151 34974714

[38] Lijuan M Xiaoli L Xiangdong D Guiqin Y Seonjoo L Yingyang Z. Cognitive impairments and low BDNF serum levels in first-episode drug-naive patients with schizophrenia. Psychiatry Res. (2018) 263:1–6. doi: 10.1016/j.psychres.2018.02.034 29482040

[39] Jiansong Z Bao-Liang Z Yu-Tao X Qiongni C Xiao–Lan C Christoph UC. Prevalence of aggression in hospitalized patients with schizophrenia in China: A meta-analysis. Asia-Pacific Psychiatry. (2015) 8:60–9. doi: 10.1111/appy.12209 26346165

[40] Everett J Lavoie K Gagnon JF Gosselin N. Performance of patients with schizophrenia on the Wisconsin Card Sorting Test (WCST). J Psychiatry Neurosci. (2001) 26:123. 11291529 PMC1407748

[41] Davidson M. Cognitive impairment as a diagnostic criterion and treatment target in schizophrenia. World Psychiatry. (2019) 18:171. doi: 10.1002/wps.20651 31059612 PMC6502436

[42] Carlberg L Schosser A Calati R Serretti A Massat I Papageorgiou K. Association study ofCREB1polymorphisms and suicidality in MDD: results from a European multicenter study on treatment resistant depression. Int J Neurosci. (2014) 125:336–43. doi: 10.3109/00207454.2014.936554 24955721

[43] Brezo J Klempan T Turecki G. The genetics of suicide: A critical review of molecular studies. Psychiatr Clinics Of North America. (2008) 31:179–203. doi: 10.1016/j.psc.2008.01.008 18439443

[44] Yan D Thomas G Daniel S Hélène M Rachael LN Eric JN. CREB modulates excitability of nucleus accumbens neurons. Nat Neurosci. (2006) 9:475–7. doi: 10.1038/nn1661 16520736

[45] David JT Cervantes MC Trosky KA Juan AS Yvon D. A neural network underlying individual differences in emotion and aggression in male golden hamsters. Neuroscience. (2004) 126:567–78. doi: 10.1016/j.neuroscience.2004.04.031 15183506

[46] Yanmei Z Chun K Haijun Y Min Y Sha W Yan W. Gene-environment interactions between CREB1 and childhood maltreatment on aggression among male Chinese adolescents. Sci Reports. (2022) 12:1326. doi: 10.1038/s41598-022-05137-7 PMC8789832 35079050

[47] Yi Y Huang Y Chen Q Yang H Li H Feng Y. Violence, neurocognitive function and clinical correlates in patients with schizophrenia. Front Psychiatry. (2022) 13:1087372. doi: 10.3389/fpsyt.2022.1087372 36741559 PMC9893505

[48] O’reilly K Donohoe G Coyle C O’sullivan D Rowe A Losty M. Prospective cohort study of the relationship between neuro-cognition, social cognition and violence in forensic patients with schizophrenia and schizoaffective disorder. BMC Psychiatry. (2015) 15:155. doi: 10.1186/s12888-015-0548-0 26159728 PMC4496853

[49] Kay SR Fiszbein A Opler LA. The positive and negative syndrome scale (PANSS) for schizophrenia. Schizophr Bull. (1987) 13:261–76. doi: 10.1093/schbul/13.2.261 3616518

[50] Buizza C Strozza C Sbravati G de Girolamo G Ferrari C Iozzino L. Positive and negative syndrome scale in forensic patients with schizophrenia spectrum disorders: A systematic review and meta-analysis. Ann Gen Psychiatry. (2022) 21:36. doi: 10.1186/s12991-022-00413-2 36088451 PMC9463849

