# Supplementary material for: Correlation study of BDNF/TrkB/CREB, violence, and cognitive function in first-episode drug-naive schizophrenia patients
Source: Front Psychiatry. 2025 Jun 4;16:1586613. doi: 10.3389/fpsyt.2025.1586613 (PMC12175090; doi:10.3389/fpsyt.2025.1586613)
Supplement: Supplementary file 1 [file Table1.doc]

**Supplementary Table 1 Primer information**

| **Primer** | **primer sequence（5’-3’）** |
| --- | --- |
| BDNF forward primer | 5’- GGCTTGACATCATTGGCTGAC -3’ |
| BDNF reverse primer | 5’- TACTGAGCATCACCCTGGAC-3’ |
| TrkB Forward primer | 5’-GTCTGAACTGATCCTGGTGGG-3’ |
| TrkB Reverse primer | 5’- GGTTAGGTGCGGCCAGATTT-3’ |
| CREB Forward primer | 5’-CAGGAGTGCCAAGGATTGAAG-3’ |
| CREB Reverse primer | 5’- CGTTACAGTGGTGATGGCAG-3’ |
| GAPDH Forward primer | 5’- GGACCTGACCTGCCGTCTAG-3’ |
| GAPDH Reverseprimer | 5’- GTAGCCCAGGATGCCCTTGA -3’ |

**The information of the Secondary antibodies**

HRP Goat Anti-Rabbit IgG (H+L) (proteintech SA00001-2 Goat 1：5000)

HRP Goat Anti-Mouse IgG (H+L) (proteintech SA00001-1 Goat 1：5000)
